# Thermogenic adipose tissue aging: Mechanisms and implications

**DOI:** 10.3389/fcell.2022.955612

**Published:** 2022-08-01

**Authors:** Graciano da Nadyellem Silva, Angelica Amorim Amato

**Affiliations:** Laboratory of Molecular Pharmacology, School of Health Sciences, University of Brasilia, Brasilia, Brazil

**Keywords:** thermogenic adipose tissue, beige adipocyte, brown adipocyte, aging, senescence

## Abstract

Adipose tissue undergoes significant anatomical and functional changes with aging, leading to an increased risk of metabolic diseases. Age-related changes in adipose tissue include overall defective adipogenesis, dysfunctional adipokine secretion, inflammation, and impaired ability to produce heat by nonshivering thermogenesis. Thermogenesis in adipose tissue is accomplished by brown and beige adipocytes, which also play a role in regulating energy homeostasis. Brown adipocytes develop prenatally, are found in dedicated depots, and involute in early infancy in humans. In contrast, beige adipocytes arise postnatally in white adipose tissue and persist throughout life, despite being lost with aging. In recent years, there have been significant advances in the understanding of age-related reduction in thermogenic adipocyte mass and function. Mechanisms underlying such changes are beginning to be delineated. They comprise diminished adipose precursor cell pool size and adipogenic potential, mitochondrial dysfunction, decreased sympathetic signaling, and altered paracrine and endocrine signals. This review presents current evidence from animal models and human studies for the mechanisms underlying thermogenic adipocyte loss and discusses potential strategies targeting brown and beige adipocytes to increase health span and longevity.

## 1 Introduction

Adaptive thermogenesis is the process of heat generation elicited to maintain body temperature when homeothermic organisms are exposed to cold and occurs through non-shivering and shivering mechanisms (Celi et al., 2015). Shivering thermogenesis is an acute and short-term response mediated by skeletal muscle contraction, leading to increased substrate oxidation and heat generation (Cannon and Nedergaard, 2004). Conversely, nonshivering thermogenesis is sustained after prolonged cold exposure in skeletal muscle and thermogenic adipocytes (Cannon and Nedergaard, 2004).

In skeletal muscle, thermogenesis occurs by uncoupling sarcoplasmic reticulum calcium-ATPase (SERCA) activity from calcium transport into the sarcoplasmic reticulum and is mediated by the protein sarcolipin (Bal et al., 2012). In adipocytes, it may be mediated by uncoupling protein 1 (UCP1) activity, which uncouples respiration from ATP synthesis (Cannon and Nedergaard, 2004), or occur through UCP1-independent mechanisms. The latter comprise futile metabolic cycles, such as (i) enhanced ATP-dependent calcium cycling by the endoplasmic reticulum Ca^+2^-ATPase 2b (SERCA2b) and ryanodine receptor 2 (RyR2) (Ikeda 2017), (ii) the futile cycle of creatinine metabolism (Kazak 2015), and (iii) the triglyceride-fatty acid futile cycling (Guan et al., 2002). Furthermore, mitochondrial uncoupling itself was described to occur independently from UCP1 through the ATP/ADP symporters SLC25A4 and SCL25A5, which mediate proton influx into the mitochondrial matrix when stimulated with N-acyl aminoacids generated by peptidase M20 domain containing 1 (PM20D1) (Long et al., 2016).

Nonshivering thermogenesis in thermogenic adipocytes is long known to play a central role in the successful adaptation of small placental mammals to cold environments by enabling the maintenance of a stable body temperature. Energy dissipation as heat is also acknowledged to be a major regulator of energy homeostasis by affecting energy expenditure, glucose (Bartelt et al., 2011; Stanford et al., 2013) and lipid metabolism (Bartelt et al., 2011), and insulin sensitivity (Bartelt et al., 2011). The latter aspect of thermogenic adipocyte function supports its role as a target to develop strategies to treat obesity and metabolic diseases and was recently fueled by the identification of functional thermogenic fat depots in adult humans (Kajimura et al., 2015; Cohen and Kajimura, 2021).

Evidence from rodent studies indicates that thermogenic adipocytes affect adipose tissue biology through anti-inflammatory, anti-fibrotic, and pro-angiogenesis actions (Cohen and Kajimura, 2021). They can also impact insulin sensitivity by mechanisms other than thermogenesis and secrete various polypeptides, referred to as batokines, lipokines such as 12,13-dihydroxy-9Z-octadecenoic acid, other small molecules, and extracellular vesicles containing miRNAs, as thoroughly reviewed elsewhere (Rodríguez et al., 2020; Cohen and Kajimura, 2021). All such actions may play a role in whole-body insulin sensitivity and energy homeostasis (Cohen and Kajimura, 2021).

## 2 The many faces of thermogenic adipocytes

**FIGURE 1:**
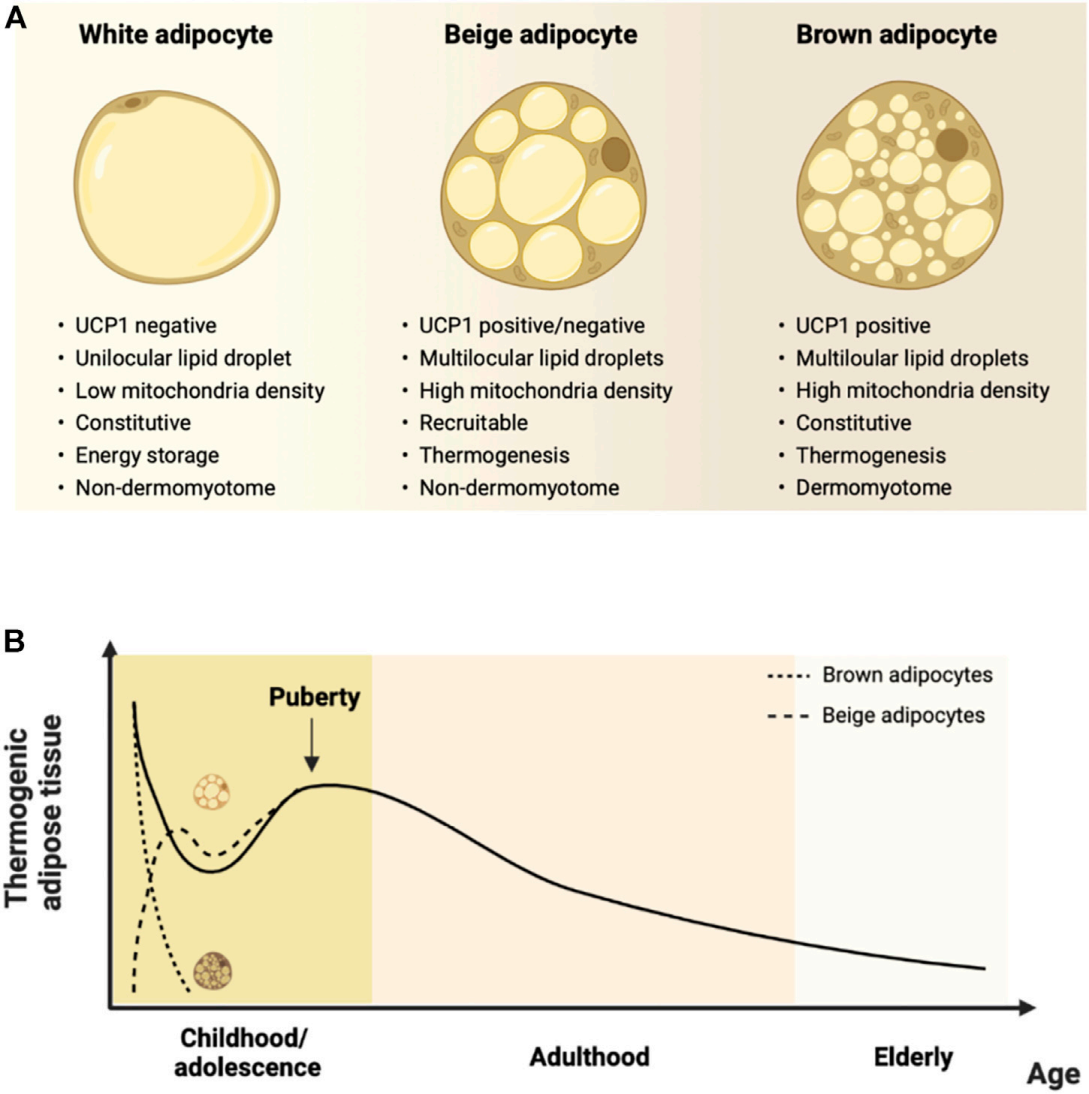
Characteristics of thermogenic adipocytes and their changes over the lifespan. **(A)**. Beige and brown adipocytes differ from white adipocytes concerning morphological and functional features. Brown adipocytes arise prenatally from a dermomyotome-derived precursor, are located within dedicated adipose depots, and are constitutive thermogenic adipocytes. Beige adipocytes have a non-dermomyotome origin, arise prenatally within white adipose depots, and are recruited by thermogenic stimuli. **(B)**. Human thermogenic adipose tissue occurrence according to age. Thermogenic adipose depots are found at birth, decline throughout infancy, and increase transiently during puberty. There is a gradual decrease during adulthood until elderly ages. Molecular studies indicate that ‘classical’ dermomyotome-derived brown adipocytes developing prenatally are found at dedicated depots and rapidly involute after birth. Conversely, non-dermomyotome-derived beige adipocytes arise postnatally within white adipose depots. Figure created with Biorender.com.

Two distinct types of thermogenic adipocytes are recognized in mammals, namely brown and beige/brite (brown-in-white) adipocytes. Both types of thermogenic adipocytes share morphological features, such as multilocular lipid droplets, mitochondria abundance, and expression of the thermogenic machinery, consistent with their ability to execute thermogenesis (Figure 1A). However, brown and beige adipocytes exhibit distinct developmental origins and functional characteristics (Ikeda et al., 2018).

Brown adipocytes arise during fetal life from myogenic factor 5 (*Myf5*) precursors in the dermomyotome and are found in distinct brown adipose tissue depots, such as the interscapular and perirenal regions of both rodents and human infants, which are densely innervated with sympathetic fibers. They constitutively express high levels of proteins comprising the thermogenic machinery and produce heat primarily by the UCP1-dependent mechanism (Ikeda et al., 2018).

Beige adipocytes develop in the postnatal period from a non-dermomyotome Myf5-negative lineage and are found within white adipose tissue depots (Cohen and Kajimura, 2021). Data from murine studies indicate a transient and extensive remodeling of white adipose tissue during the first 3 weeks of life, characterized by the emergence of beige adipocytes, as indicated by increased UCP1 expression in different depots. This peaks at around postnatal day 20 and is reversed at day 30 in C57BL6 mice, and seems to be dependent upon the interaction of genetic and environmental factors and has been thoroughly reviewed elsewhere (Bruder and Fromme, 2022).

After early postnatal life, beige adipocytes disappear or become dormant but can be recruited during adult life (Xue et al., 2007). This process is referred to as ‘browning’ or ‘beiging’ of white adipose tissue. It occurs in response to so-called ‘beiging stimuli,’ such as cold exposure, catecholamines/β3-adrenergic signaling, exercise, and thiazolidinediones (Cohen and Kajimura, 2021).

Recruitment of beige adipocytes may occur by differentiation from progenitor cells (‘*de novo* biogenesis’) or by white-to-beige conversion (transdifferentiation) of phenotypically mature white adipocytes (Barbatelli et al., 2010). The relative contribution of each pathway varies with the thermogenic stimulus (Shao et al., 2019). Beige adipocyte progenitor cells comprise a distinct cellular pool within the stroma of white adipose tissue and differentiate in response to beiging signals (Wang et al., 2013). White-to-beige conversion is a bidirectional process in which the thermogenic stimulus promotes conversion into the beige phenotype (Barbatelli et al., 2010). After its withdrawal, ‘whitening’ or beige-to-white conversion through mitochondrial clearing by autophagy occurs (Altshuler-Keylin et al., 2016). Whitened beige adipocytes become dormant but can regain their thermogenic features following a subsequent thermogenic stimulus (Altshuler-Keylin et al., 2016). Given that clonal analysis of adipogenic precursor cells indicate that beige and white adipocyte precursors are distinct, the current view is that the phenotypically white adipocytes that ‘transdifferentiate’ into beige adipocytes are, in fact, dormant beige adipocytes (Wu et al., 2012; Shinoda et al., 2015; Xue et al., 2015).

The early postnatal pool of beige adipocytes, remaining dormant but being recruitable throughout life, seems to be a critical determinant of beiging capacity during adult life (Cohen and Kajimura, 2021). Factors determining the size of beige adipocyte pool established in early life are yet to be characterized but may involve genetic background, epigenetic changes, and environmental factors. In a study assessing human newborn thermogenic adipose tissue by non-invasive infrared thermography in the interscapular area, mild cold exposure was associated with increased interscapular temperature. It was accompanied by increased bone morphogenic protein 8B (BMP8B) circulating levels but not by other well-acknowledged thermogenesis-related hormones, such as FGF21 and thyroid hormone (Urisarri et al., 2021). Although the exact identity of thermogenic adipocytes, brown or beige, was not determined, the findings point to the possible role of cold exposure and BMP8 in the postnatal development of the thermogenic program.

Interestingly, the presence of breast milk-specific lipid species (alkylglycerol-type, AKG, ether lipids) was recently described in the adipose tissue of humans and mice. In mice, it was reported that AKG increased the appearance of beige adipocytes within white adipose tissue until late infancy by the interleukin 6/STAT3 pathway. In contrast, lack of AKG intake in infancy led to premature loss of beige adipocytes and increased white adipose tissue mass (Yu et al., 2019). Therefore, breast milk AKG may affect the beige adipocyte precursor pool size established in and may mediate the protection from obesity later in life conferred by breastfeeding.

Data from recent studies involving cellular lineage determination and single-cell transcriptomics suggest there are different subtypes of brown and beige adipocytes, arising from distinct progenitors and comprising adipocyte populations with specific functional features (Cohen and Kajimura, 2021). In mouse interscapular brown adipose tissue depots, two subtypes of adipocytes were described according to the expression level of adiponectin (Song et al., 2020). Brown adipocytes expressing low levels of adiponectin express endothelial markers and have larger lipid droplets, lower levels of thermogenic genes, and lower mitochondrial content compared to those expressing high levels of adiponectin (Song et al., 2020).

Two types of beige adipocytes were identified so far (Cohen and Kajimura, 2021). The classical beige adipocyte arises from αSMA^+^ and PDGFRα^+^ adipose progenitor cells. It expresses the thermogenic machinery in a β-adrenergic-dependent fashion and uses the energy from fatty acid oxidation to generate heat (Kajimura et al., 2015). More recently, a second type of beige cell was described, the so-called glycolytic beige adipocyte (g-beige adipocyte) (Chen et al., 2019). The latter arise from myogenic MYOD^+^ and PDGFRα^+^ progenitors in response to acetylcholine produced by immune cells in inguinal fat and independently from β-adrenergic signaling (Jun et al., 2020). g-beige adipocytes use glucose oxidation as their primary fuel for thermogenesis (Chen et al., 2019).

Although anatomical evidence for the presence of thermogenic adipose tissue throughout life has been long known (Heaton, 1972), the interest in thermogenic fat as a target to treat metabolic disease was fueled over the last two decades owing to the identification that it is functionally active in adult humans, as indicated by 18F-fluorodeoxyglucose positron emission tomography (PET/CT) combined with computed tomography (Hany et al., 2002). Notably, the presence of thermogenic fat was positively correlated with lower body mass and improved measures of metabolic health in humans and negatively correlated with age (Cypess et al., 2009; van Marken Lichtenbelt et al., 2009; Virtanen et al., 2009).

Thermogenic adipose depots in adult humans were initially identified in the supraclavicular area (Cypess et al., 2009; van Marken Lichtenbelt et al., 2009; Virtanen et al., 2009) and subsequently in cervical, axillary, mediastinal, abdominal, and paravertebral regions (Leitner et al., 2017). They were initially referred to as brown adipose tissue, but it is currently acknowledged that they are heterogeneous and comprise both brown and beige adipocytes. Even within a distinct adipose depot, such as the supraclavicular depot, multiple populations of thermogenic adipocytes can be found (Wu et al., 2012; Lidell et al., 2013; Shinoda et al., 2015). Conversely, in human subcutaneous white adipose tissue, recruitable thermogenic fat has a molecular signature consistent with beige adipocytes (Leitner et al., 2017; Finlin et al., 2018), indicating that the latter may be the exclusive or predominant thermogenic adipocyte in subcutaneous fat depots.

Hence, current evidence supports that ‘classical’ brown adipose tissue, comprising cells of dermomyotome origin, is found in human infants in dedicated fat depots (Cohen and Kajimura, 2021). In contrast, thermogenic adipocytes contain a mixed cell population of brown and non-dermomyotome-derived beige cells during postnatal life (Cohen and Kajimura, 2021). Conversely, rodents maintain discrete brown adipose tissue during adulthood, and beige adipocytes emerge postnatally within white adipose tissue depots. However, there is an overall age-associated decline in thermogenic fat activity in both rodents and humans (Cohen and Kajimura, 2021).

## 3 Thermogenic adipose tissue changes in aging

Aging is associated with profound changes in anatomical and functional aspects of adipose tissue with varying magnitude and observed even in healthy individuals. There is an increase in overall fat mass (Reinders et al., 2017) and redistribution characterized by a decrease in the proportion of subcutaneous adipose tissue and thermogenic adipose tissue in the cervical, supraclavicular, and superior mediastinal regions, with a concomitant increase in the proportion of visceral and bone marrow adipose tissue (Mancuso and Bouchard, 2019). Anatomical changes are accompanied by dysregulation in the secretion of adipokines, lipid metabolism, central carbon metabolism, electron transport chain complexes, and inflammation (Yu et al., 2020). These, in turn, may reduce the ability of adipose tissue to buffer excess nutrients and lead to a pro-inflammatory and insulin-resistant state, ultimately increasing the risk of obesity and metabolic diseases (Ouchi et al., 2011). Therefore, age-related adipose tissue remodeling and its consequent dysfunction are closely related to the development of diseases that are more prevalent with aging. Importantly, obesogenic conditions may synergize with age-related adipose tissue dysfunction to increase the risk of metabolic disorders (Conte et al., 2019) or possibly recapitulate those abnormalities at an earlier stage.

Changes in thermogenic adipose tissue structure and function across the lifespan have been more extensively investigated in animal models (Bruder and Fromme, 2022). Investigation more complex in humans and currently based on anatomical and functional imaging by nuclear magnetic resonance and PET/CT imaging, histological sampling of accessible adipose tissue sites, or postmortem studies (Kajimura et al., 2015; Cohen and Kajimura, 2021).

In fetal and early postnatal life, thermogenic adipose tissue in humans is found within the interscapular, supraclavicular, cervical, axillary, mediastinum, perirenal, and other abdominal depots, as indicated by histological and magnetic resonance imaging analysis (Aherne and Hull, 1964; Sharp et al., 2012). It is critical for thermoregulation at birth since shivering thermogenesis is not fully developed in newborns (Zoico et al., 2019). Findings from studies conducted using PET/CT imaging (Sharp et al., 2012) or assessing UCP1 expression (Lean et al., 1986) indicate that thermogenic adipose tissue activity decreases in the interscapular region shortly after birth (Sharp et al., 2012). However, it is found in other locations (Gilsanz et al., 2012; Sharp et al., 2012) during childhood and is positively correlated with the volume of cervical musculature (Gilsanz et al., 2012).

During adolescence, there is a significant increase in both the activity and volume of thermogenic adipose tissue from early to late puberty, most prominently in boys than in girls and which is positively correlated with abdominal musculature volume (Gilsanz et al., 2012) and femoral cross-sectional and cortical bone area (Ponrartana et al., 2012). In adults, thermogenic adipose tissue is detected in the cervical, supraclavicular, axillary, mediastinal, paraspinal, and abdominal (including subcutaneous) regions (Leitner et al., 2017), more frequently in women than men, and declines progressively with age (Cypess et al., 2009; Pfannenberg et al., 2010).

The identity of thermogenic adipocytes throughout development has been extensively examined in rodents. ‘Classical’ dermomyotome-derived brown adipocytes develop prenatally and are found at discrete adipose tissue depots in the interscapular, subscapular, cervical, and perirenal regions (Fitzgibbons et al., 2011; Waldén et al., 2012). The largest brown adipose tissue depot is in the interscapular area (Cohen and Kajimura, 2021) and persists throughout life, as opposed to what is observed in humans (Sharp et al., 2012). Non-dermatomyotome-derived beige adipocytes develop postnatally and are predominantly found within subcutaneous white adipose tissue depots in the suprascapular, anterior abdominal, and inguinal regions (Zhang et al., 2018; Cohen and Kajimura, 2021).

The molecular identity of human thermogenic adipocytes has also been examined by assessing the expression of brown and beige-selective molecular markers identified in rodent adipose tissue (Wu et al., 2012). Different studies reported that most adult thermogenic fat depots display a molecular signature consistent with the presence of brown and beige adipocytes, with the latter being predominant. In contrast, thermogenic adipocytes recruited within subcutaneous white adipose tissue possess the molecular markers of beige adipocytes (Cypess et al., 2009; Wu et al., 2012). Moreover, the predominance of brown or beige adipocytes varies within different adipose tissue depots and different regions of a single depot (Cypess et al., 2013). It was reported that neck adipose depots from healthy adult humans expressed linear marker genes of white adipocytes at superficial locations, beige adipocytes at intermediate locations, and brown adipocytes at deeper areas (Cypess et al., 2013).

Notably, the view that the predominant adult human thermogenic adipose tissue resembles more closely mouse beige adipocytes was recently challenged. Mouse studies conducted in ‘physiologically humanized’ conditions (middle-aged mice fed a high-fat diet and housed at thermoneutrality, 30°C) as opposed to ‘standard’ conditions (young mice fed chow diet and housed at standard temperature, 20°C) indicate that classical brown adipose tissue exhibits molecular markers of human thermogenic adipose tissue (Cannon et al., 2020).

Despite the controversy regarding the comparative phenotype of thermogenic adipocytes in mice and humans, current evidence indicates a gradual decline in thermogenic adipose tissue mass and activity after puberty (Figure 1B). In humans, there is an apparent plateau around the sixth decade of life, followed by a subsequent decrease (Persichetti et al., 2013). The decline is earlier at peripheral sites and occurs at older ages in deep locations, such as in the perirenal or perivascular areas (Pfannenberg et al., 2010; Rogers, 2015). It is notably accompanied by increased lipid accumulation, reduced mitochondrial and UCP1 content, and reduced ability to recruit beige adipocytes (Rogers, 2015; Berry et al., 2017; Gonçalves et al., 2017). The latter features are viewed as an age-related ‘whitening’ of thermogenic adipose tissue and are consistent with the reduction in nonshivering thermogenesis (McDonald et al., 1988; Scarpace et al., 1992; Florez-Duquet and McDonald, 1998; McDonald and Horwitz, 1999; Saito et al., 2009) and increased risk of cold-induced hypothermia with aging (Florez-Duquet and McDonald, 1998). The age-associated changes in thermogenic adipose tissue may also be mechanistically linked to the increase in the age-associated risk of metabolic diseases (Ou et al., 2022).

## 4 Mechanisms of thermogenic adipose tissue decline with aging

**FIGURE 2:**
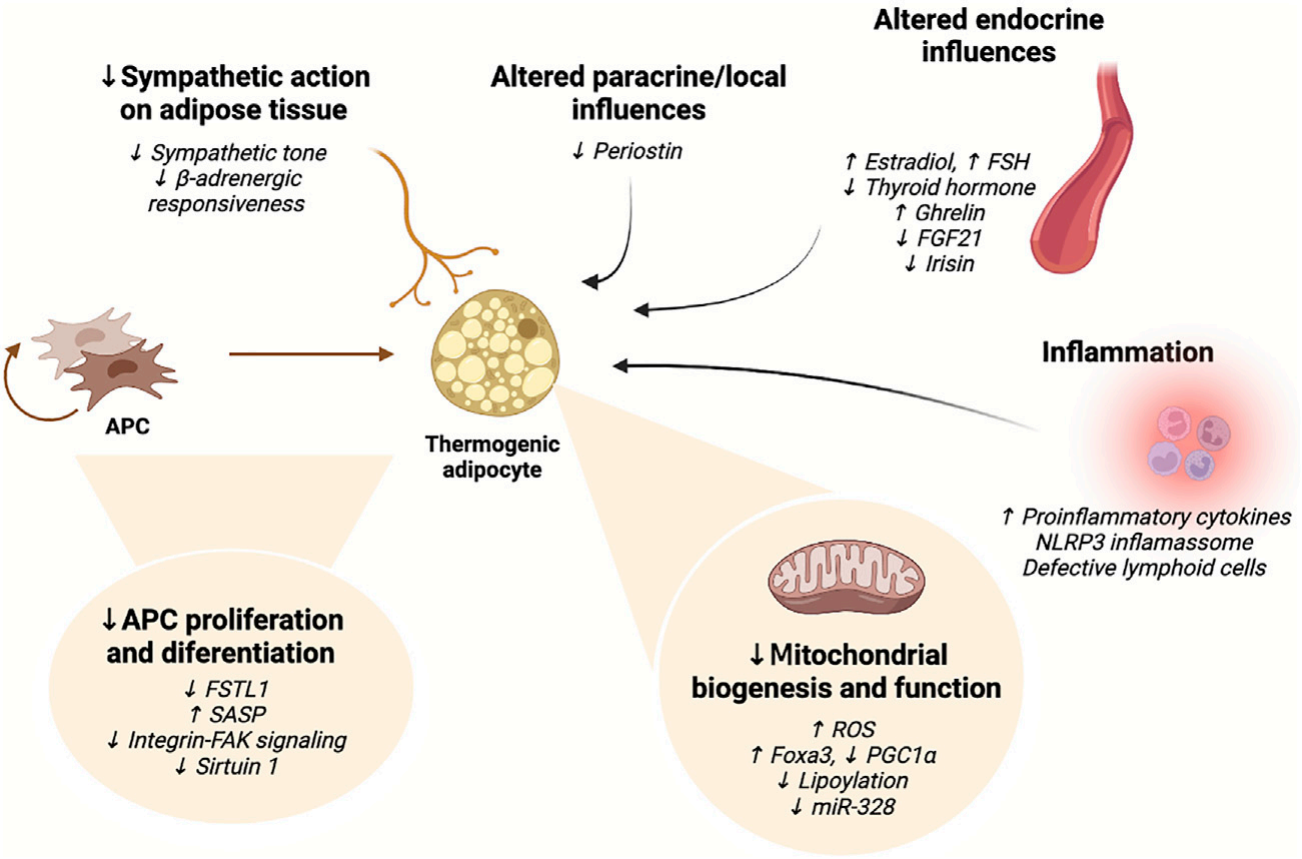
Summary of the mechanisms currently implicated in the age-related impairment of thermogenic adipocyte activity. Aging results in impaired ability of thermogenic adipocyte precursor cells to proliferate and differentiate, reduced mitochondrial biogenesis, mitochondrial dysfunction, and diminished sympathetic action. Inflammatory signaling negatively affects brown and beige adipocyte differentiation and impairs thermogenic activity. Moreover, various endocrine and paracrine influences on adipocytes change with advanced age, also impacting thermogenic adipocyte function. FAK: focal adhesion kinase; FGF21: fibroblast growth factor 21; FSH: follicle-stimulating hormone; FSTL1: follistatin-like one; miR: microRNA; NLRP3: NACHT, LRR, and PYD domains-containing protein three; PGC1α: peroxisome proliferator-activated receptor gamma coactivator-1 alpha; ROS: reactive oxygen species; SASP: senescence-associated secretory phenotype. Figure created with Biorender.com.

Current evidence indicates that a myriad of mechanisms is implicated in the decline of thermogenic adipose tissue mass and function with aging, which affect the number and ability of precursor cells to differentiate into thermogenic adipocytes and their thermogenic capacity, as summarized in Table 1 and Figure 2.

**TABLE 1:** Mechanisms underlying age-related thermogenic adipocyte dysfunction.

Age-related change	Reported mechanism(s)	Organism, adipocyte type	References
Adipose precursor cell proliferation and differentiation impairment	*↓ FSLT1* expression: impaired recruitment	Rabbit, brown adipocyte	Huang et al. (2022)
	↓ αV/β1 and αV/β5 integrin-FAK signaling: impaired APC proliferation and recruitment	Mouse, beige adipocyte	Oguri et al., 2020
	↑ Senescence-associated secretory program: impaired recruitment	Mouse, beige adipocyte	Tajima et al. (2019)
	↓ Sirtuin 1 levels and ↑ p53/p21 pathway activity: impaired recruitment	Human AT-MSC, beige adipocyte	Khanh et al. (2018)
Adipose tissue-intrinsic mechanisms			
Impaired mitochondrial biogenesis and function	↑ Foxa3: CREB1-mediated suppression of PGC1α: ↓ mitochondrial biogenesis	Mouse, brown and beige adipocytes	Bagattin et al. (2010)
	↓ Mitochondrial lipoylation due to ↓ iron-sulfur cluster formation: ↓ mitochondrial function	Mouse, brown adipocyte	Tajima et al. (2019)
	↓ mIR-328: ↓ mitochondrial function	Mouse, brown adipocyte	Oliverio et al. (2016)
Extracellular matrix	↓ Periostin: ↓ mitochondrial function	Mouse, brown and (?) beige adipocyte	Graja et al. (2018)
Decreased sympathetic action	↓ Sympathetic tone	Human (functional imaging studies)	Bahler et al. (2016)
	↓ β-adrenergic receptor density	Rat, brown adipocyte	Scarpace et al. (1988)
Altered paracrine and endocrine influences	↓ ER signaling	Mouse, brown adipocyte	Kaikaew at al., 2021
	↑ FSH	Mouse, brown and beige adipocytes	Liu et al. (2017)
	↓ TH signaling (?)	Mouse, brown and beige adipocytes	Weiner et al. (2017)
			Gauthier et al. (2020)
	↑ Ghrelin/GHSR	Mouse, brown adipocyte	Lin et al. (2011)
	↓ Irisin (?)	Mouse, brown and beige adipocytes	Kwon et al. (2020)
Immune cells and inflammation	↑ Proinflammatory cytokine action	Mouse, brown and beige	Goto et al. (2016)
	↑ NLRP3 inflammasome: catecholamine degradation	Mouse, (?)	Camell et al. (2017)
	Defective type 2 innate lymphoid cells	Mouse, defective cold response	Goldberg et al. (2021)

AT-MSC, adipose-derived mesenchymal stem cell; CREB, cAMP-responsive element binding protein one; ER, estrogen receptor; Foxa3, transcription factor forkhead box protein A3; FSH, follicle-stimulating hormone; GHSR, growth hormone secretagogue receptor (ghrelin receptor); PGC1α, PPARγ, coactivator one alpha; TR, thyroid hormone (?) Indirect evidence from studies indicated age-related associated changes in the described factors and independent studies indicating the role of the factor in thermogenic adipocyte function.

### 4.1 Decreased thermogenic adipocyte progenitor cell proliferation and differentiation

Human brown adipose tissue involutes early in postnatal life, differently from mouse tissue. In mice, brown adipocyte progenitor cells (APC) remain in the brown adipose tissue throughout life and are essential for tissue regeneration after damage (Moser et al., 2021). They can also contribute to a certain extent to brown adipocyte recruitment in response to thermogenic stimuli (Sanchez-Gurmaches and Guertin, 2014; Lee et al., 2015), even when mice are maintained under ‘physiologically humanized’ conditions (thermoneutrality with high-fat diet feeding) (Razzoli et al., 2016).

The maintenance of brown adipose tissue throughout life in mammals is positively correlated with the surface-to-volume ratio since it is higher in mice and decreases progressively from rats (Florez-Duquet and McDonald, 1998) to rabbits (Huang et al., 2022) and humans (Huang et al., 2022). Therefore, findings from mouse models may not reflect aging-related changes in human brown adipose tissue. Huang et al. (2022) recently provided novel insights into brown adipocyte aging using a rabbit model, which resembles human brown adipose tissue biology more closely than mice. By using single-cell RNA sequencing, they showed that rabbit and human interscapular brown adipose tissue APC express high levels of the *FSTL1* gene, encoding the follistatin-like one glycoprotein, which could act to maintain their renewal capacity and differentiation competence. In parallel with tissue involution in rabbits, there is decreased brown APC expression of *FSTL1* and reduced competence or ability to differentiate into brown adipocytes upon β3-adrenergic activation. Conversely, mice retain *FSTL1* constitutive expression at later life stages, and its genetic ablation recapitulates the brown adipocyte involution observed in rabbits and humans (Huang et al., 2022).

Beige APC emerge postnatally in the vascular stroma of different white adipose tissue depots (Wu et al., 2012; Shinoda et al., 2015; Min et al., 2016). They comprise a heterogeneous cellular population expressing *Acta2* (encoding αSMA), *Pax3*, *Pdgfra*, or *Pdgfrb* (Lee Y. H. et al., 2012; Sanchez-Gurmaches and Guertin, 2014; Berry et al., 2016; Vishvanath et al., 2016), that is distinct from embryonic brown APC and white APC (Wu et al., 2012; Shinoda et al., 2015). The beige APC pool is maintained during postnatal life by its ability to proliferate and responds to beiging signals by differentiating into beige adipocytes (*de novo* beige biogenesis). Notably, aging is associated with a significantly decreased ability of beige APC to proliferate and differentiate (Khanh et al., 2018; Tajima et al., 2019), possibly mediating the aging-associated reduction of beige biogenesis.

The mechanisms underlying the reduced proliferative potential of brown and beige APC remain largely unexplored. Findings from a recent study employing single-cell RNA-sequencing identified CD81 as a surface marker of beige APC. Notably, CD81 was shown to be actively involved in *de novo* beige adipocyte biogenesis following cold exposure by complexing with αV/β1 and αV/β5 integrins and activating integrin-FAK signaling. CD81^+^ APC displayed down-regulation of senescence pathway-related pathways and high proliferative capacity. However, CD81^+^ APC proliferation decreased with aging (Oguri et al., 2020).

Emerging evidence points to the interaction between cellular senescence pathways and APC proliferation. Beige αSMA^+^ APC progenitors from inguinal white adipose tissue from old (6-months) mice and human stromal vascular cells from the abdomen and hip of more senior individuals were shown to exhibit an overall senescence-like phenotype. Such phenotype includes upregulation of genes related to cell cycle arrest and induction of the senescence-associated secretory program consisting of cytokines, chemokines, proteases, and growth factors (Coppé et al., 2008). In mouse cells, the latter findings were accompanied by decreased ability to recruit beige adipocytes in response to cold (Tajima et al., 2019).

Decreased differentiating ability of beige APC was confirmed in a different study involving human cells, which further implicated aging-associated reduced sirtuin 1 levels as a possible underlying mechanism, given that overexpression of sirtuin 1 in beige APC was able to recover cells from the senescent phenotype by impairing the p53/p21 pathway (Khanh et al., 2018). Activation of the latter pathway is critical for senescence regulation, given its role in mediating cell cycle arrest by regulating the expression of anti-proliferative genes (Kumari and Jat, 2021).

It is also possible that trophic factors from the circulation or in the adipose tissue microenvironment mediate the changes in the proliferative potential of thermogenic APC. In culture, brown preadipocytes from old (26-month-old) F344 rats were shown to exhibit a comparable proliferative response to serum-supplemented culture medium when compared with their young (6-month-old) counterparts (Florez-Duquet and McDonald, 1998).

It is currently unknown whether aging affects the proportion of different subpopulations of thermogenic adipocytes by differential effects on their precursors. It was recently shown that brown adipocytes expressing high levels of adiponectin declined gradually in aged mice, as opposed to those expressing lower levels of adiponectin (Song et al., 2020).

### 4.2 Adipose tissue-intrinsic mechanisms

Events intrinsically related to thermogenic adipocytes that could mediate the age-related decline in their thermogenic capacity are cellular senescence affecting APC, as previously discussed, and those related to mitochondrial function. Thermogenic adipocytes are rich in cristae-dense mitochondria crucial for UCP1-dependent heat production. They are maintained by constitutive mitochondrial biogenesis in brown adipocytes. Conversely, in beige adipocytes, mitochondrial content increases upon thermogenic stimulation, which signals for mitochondrial biogenesis, and decreases when the latter is withdrawn by autophagy-mediated clearance (Ikeda et al., 2018).

Aging is associated with global mitochondrial dysfunction (López-Otín et al., 2013), most likely stemming from several molecular events, including decreased mitochondrial oxidative phosphorylation, leading to increased lipotoxic and glucotoxic reactions, or increased levels of reactive oxygen species and oxidative damage (Amorim et al., 2022). Such events, in turn, result in mitochondrial DNA mutations that accumulate and promote a vicious cycle of increased reactive oxygen species production, ultimately leading to mitochondrial dysfunction (Amorim et al., 2022). Throughout the lifespan, there is also diminished mitochondrial content (Valle et al., 2008a) and biogenesis (López-Otín et al., 2013) and significantly decreased expression and activity of thermogenesis-related mitochondrial proteins, including UCP1 (Valle et al., 2008a; Zoico et al., 2019). The mechanisms underlying the changes in thermogenic adipocyte mitochondrial activity have been explored in some studies.

The transcription factor forkhead box protein A3 (Foxa3) was previously described to mediate high-fat diet-induced expansion of visceral white adipose tissue and was recently implicated in decreasing thermogenic adipose tissue activity with aging. It was shown that Foxa3 is upregulated in mouse brown and inguinal white adipose depots during aging and suppresses cAMP-responsive element binding protein 1-regulated transcription of PGC1α, a master regulator of mitochondrial biogenesis (Bagattin et al., 2010). Foxa3 knockout or overexpression in inguinal white adipose tissue confirmed its direct involvement in modulating beige adipocyte recruitment and function. Interestingly, Foxa3-null mice exhibited increased beige adipocyte content, mitochondrial thermogenic capacity, and longevity (Ma et al., 2014).

Mitochondrial proteomic analysis of mouse interscapular brown adipose tissue revealed that aging led to diminished fuel oxidation ability, determined by reduced levels of mitochondrial lipoylation due to reduced mitochondrial iron-sulfur cluster formation required for maturation of lipoate-containing proteins in mitochondria. The latter finding was shown to be directly implicated in the impairment of glucose catabolism and thermogenic activity in this adipose depot (Tajima et al., 2019).

An additional mechanism involved in thermogenic adipose tissue aging is related to the ubiquitin-proteasomal pathway (Bartelt et al., 2011). It was recently shown that activation of brown adipose tissue thermogenic activity following cold exposure upregulated the endoplasmic reticulum-embedded transcription factor nuclear factor erythroid 2-like 1 (Nfe2l1 or nuclear respiratory factor 1, Nrf1), which in turn increased proteasomal activity in this adipose depot. Conversely, brown-adipocyte-specific knockout of *Nfe2l1* led to an impaired cold response, as indicated by lower mitochondrial content, abnormal thermogenic function, and whitening of brown adipose tissue (Bartelt et al., 2011). Nfe2l1 expression is regulated by transcription coactivators, being PGC1α the most studied one, and regulates the expression of the enhancer protein TFAM (transcription factor A, mitochondrial), which ultimately stimulates mitochondrial DNA duplication. The Nfe2l1-TFAM pathway is implicated in PGC1α mediated mitochondrial biogenesis, as has been thoroughly reviewed elsewhere (Gureev et al., 2019). Both mitochondrial dysfunction and diminished proteostasis are considered hallmarks of aging (López-Otín et al., 2013). Interestingly, Nfe2l1 expression was shown to decrease in the heart of old rats when compared with their young counterparts (Viña et al., 2009). Whether this also applies to adipose tissue remains to be investigated but could causally be linked to age-related impairment in thermogenic adipose tissue function.

Changes in mitochondrial thermogenic function with aging may also be related to diminished expression of microRNAs in adipose tissue. It was reported that progeroid *Ercc1* knockout mice exhibited reduced expression of the microRNA-processing enzyme Dicer1, accompanied by down-regulation of microRNA levels when compared with the long-lived homozygous Ames dwarf mutant mice. Further analysis indicated that miR-328 was a key regulator of brown adipocyte differentiation and its overexpression in primary immortalized brown adipocytes increased mitochondrial proteins related to thermogenesis and mitochondrial thermogenic activity (Oliverio et al., 2016). Whether miR-328 also affects mouse beige adipocyte mitochondrial function or its human ortholog impacts thermogenic adipocytes remains to be investigated.

In addition to adipocyte-intrinsic mechanisms, other factors in the adipose tissue microenvironment may play a role in thermogenic adipocyte changes with age. There is evidence that this is the case for immune-inflammatory cells resident within the adipose tissue, and it is not possible to rule out other factors, such as microRNAs, nonprotein soluble factors, growth factors, and extracellular matrix molecules may also play a role. Accordingly, it was shown that aging was accompanied by downregulation of the matricellular protein periostin in mouse brown and white adipose tissue, and germline periostin gene knockout was associated with impaired response to both cold and pharmacological adrenergic stimulation (Graja et al., 2018).

### 4.3 Impairment of sympathetic input to thermogenic adipocytes

The sympathetic nervous system innervates brown and white adipose tissue and is, to a great extent, critical for the recruitment and activation of thermogenic adipocytes. Sympathetic activation results in catecholamine release in response to cold exposure, which activates β3-adrenergic receptors in mature thermogenic adipocytes. This leads to increased lipolysis, UCP1 expression and activity, and other processes involved in thermogenic activity. Furthermore, there is evidence pointing that beige APC express β1-adrenergic receptors, through which catecholamine signaling induces proliferation and differentiation (Bronnikov et al., 1992; Jiang et al., 2017).

Diminished sympathetic tone to adipose tissue or its responsiveness with aging could be implicated in the decline in cold-induced thermogenic adipose tissue recruitment and activity (and resultant decline in nonshivering thermogenesis) that is observed in both rodents (McDonald and Horwitz, 1999) and humans (Saito et al., 2009; Yoneshiro et al., 2013). Data from rodent studies suggest that the sympathetic tone to thermogenic adipose tissue is not decreased with aging. Indeed, 30-month-old mice exhibited higher sympathetic tone than 10-month-old mice, both basally and in response to cold exposure, as indicated by monitoring the electrical activity of intercostal nerve entering interscapular brown adipose tissue (Kawate et al., 1993). Further evidence supporting that tissue responsiveness but not stimulation declines with aging is the report of diminished β-adrenergic receptor density in rat brown adipose tissue (Scarpace et al., 1988). However, it is still not clear whether reduced sympathetic sensitivity is an adipocyte-intrinsic event or the consequence of other aging-related processes.

In contrast to the findings from rodent studies, human studies using the radiolabeled norepinephrine analog 123I-metaiodobenzylguanidine combined with PET/CT imaging indicated that older subjects exhibit both reduced thermogenic adipose tissue activity and decreased sympathetic drive when compared with their younger counterparts, independently from body mass (Bahler et al., 2016). Therefore, the reduced sympathetic drive may impair thermogenic adipose tissue activation and the response to cold, which are observed with aging in humans. The reasons for the discrepant findings when comparing rodents and humans are not defined but could be related to the different methods employed to examine sympathetic activity. Moreover, studies involving mice and rats examined dedicated brown fat depots, whereas, in adult humans, current evidence indicates that beige adipocytes predominantly comprise thermogenic adipose tissue. It is not possible to exclude that different thermogenic adipocyte populations could exhibit distinct patterns of sympathetic stimulation changes with age.

### 4.4 Altered local and endocrine influences on thermogenic adipocytes

Aging is associated with several endocrine changes that may affect thermogenic adipose tissue, including diminished gonadotropic and thyrotropic axis function. Estrogen and androgen receptors are expressed in human (Velickovic et al., 2014) and mouse (Rodríguez-Cuenca et al., 2007) fetal brown adipose tissue depots. Moreover, serum levels of 17β-estradiol were shown to decline during aging in rats and correlate with reduced expression of thermogenesis-related mitochondrial proteins in male and female rats (Valle et al., 2008b). In humans, a recent investigation using infrared thermography of the supraclavicular region indicated that women had a greater thermogenic response to cold exposure than men, which was associated with circulating levels of 17β-estradiol (Fuller-Jackson et al., 2020).

The actions of sex steroids on brown adipocytes have been thoroughly reviewed elsewhere (Kaikaew et al., 2021). There is evidence that estrogen receptor signaling stimulates brown adipocyte differentiation and activity by direct actions on adipose tissue and indirect effects through a hypothalamic action that leads to increased sympathetic drive (Kaikaew et al., 2021). Androgen actions on brown adipocytes are not fully established, and it is unknown whether it is mediated through direct effects, hypothalamic mechanisms, or even aromatization into estrogens (Kaikaew et al., 2021).

Likewise, the effects of progesterone on brown adipocytes and its underlying mechanisms remain to be established and could be mediated by progesterone, glucocorticoid, or even mineralocorticoid receptors (Kaikaew et al., 2021). Despite much-needed research to understand the exact mechanisms of sex steroid action on brown adipocyte differentiation and activity, such hormones could account for the sexual dimorphism observed until women become postmenopausal (Kaikaew et al., 2021). The expression pattern of sex steroid receptors and their actions on beige adipocytes within white adipose tissue is less well established (Bernasochi et al., 2019).

The impact of age-related changes in gonadotropic axis function may also be associated with increased circulating levels of the gonadotropin follicle-stimulating hormone (FSH). Blocking FSH action in mice with a polyclonal antibody was shown to reduce adiposity, induce beige adipocyte recruitment, activate brown adipose tissue, and augment thermogenesis. These effects were recapitulated in heterozygous FSH receptor knockout mice (Liu et al., 2017).

Conversely, corticotropic axis function does not change considerably with age, and glucocorticoid receptor signaling in adipose tissue is well-known for its actions to impair brown adipose tissue (and possibly also beige adipocytes) thermogenic function (Soumano et al., 2000). It is therefore hypothesized that reduced thermogenic adipocyte activity with aging could be the result from an imbalance between the decreased stimulatory effects of estrogens and maintained action of glucocorticoids (Nedergaard and Cannon, 2010).

Thyroid hormone is a critical regulator of thermogenic adipocyte recruitment and activity, affecting brown and beige adipocytes in rodent models and humans (Lee J. Y. et al., 2012; Weiner et al., 2017). Aging is associated with slightly higher circulating TSH levels and lower T4 secretion, but with reduced activity of type 1 and type 2 deiodinases and reduced expression of thyroid hormone transporter in some tissues (Gauthier et al., 2020). Aging was also shown to decrease the lipogenic action of thyroid hormone in the liver and epidydimal and retroperitoneal white adipose depots (Levacher et al., 1988). Therefore, it is plausible that reduced thyroid hormone secretion and diminished sensitivity to thyroid hormone, possibly mediated by downregulation of type 2 deiodinase activity or reduced cellular uptake of thyroid hormone, may contribute to reduced thermogenic adipose tissue function in older individuals.

Ghrelin receptor (growth hormone secretagogue receptor) is highly expressed in the hypothalamus and at peripheral sites, including adipose tissue (Camilleri et al., 2009). Ghrelin is a gut-derived orexigenic peptide hormone that functions as a critical component of the gut-brain axis for energy homeostasis regulation (Camilleri et al., 2009). Both ghrelin circulating levels and the expression of its receptor increase with aging in mice (Sun et al., 2007), and ghrelin signaling was reported to directly impair brown adipose tissue activity and decrease energy expenditure (Lin et al., 2011). Notably, ghrelin receptor expression was detected only in mouse visceral white adipose tissue, and germline ghrelin receptor knockout did not affect beige adipocytes (Lin et al., 2011). These findings implicate ghrelin as a possible mediator of age-associated changes in brown adipocytes, at least in mice. Whether this also applies to humans remains to be established.

Fibroblast growth factor 21 (FGF21) is primarily secreted by the liver, acting as an endocrine factor or hepatokine, and has also been shown to be produced in adipose tissue, with paracrine and endocrine actions (Cuevas-Ramos et al., 2019). FGF21 acts centrally and peripherally to increase insulin sensitivity, favorably affect glucose and lipid metabolism, and increase thermogenesis (Cuevas-Ramos et al., 2019). The latter effect is mediated activation of brown adipose tissue and recruitment and activation of beige adipocytes by direct actions on adipose tissue (Fisher et al., 2012) and central actions to increase sympathetic activity (Owen et al., 2014). Interestingly, aging is associated with a progressive increase in FGF21 circulating levels in rodents and humans, independently of body composition, suggesting a state of FGF21 resistance (Villarroya J. et al., 2018). However, in a study involving healthy aged individuals, no changes were observed in adipose tissue responsiveness to FGF21 ‘*ex vivo*’ (Villarroya J. et al., 2018). It cannot be excluded that a ‘pre-receptor’ factor *in vivo* could mediate an FGF-resistant state in aged subjects.

Several skeletal muscle-derived factors referred to as myokines (or exerkines when released in response to exercise), may mediate the favorable effects of exercise on health span and longevity (Chow et al., 2022). Some myokines have been shown to regulate thermogenic adipose tissue development and activity, such as irisin, the secreted fragment from the membrane protein FNDC5 (Boström et al., 2012), meteorin-like (Rao et al., 2014), and interleukin 6 (Knudsen et al., 2014). Irisin secretion decreases with age (Kwon et al., 2020) and may, therefore, plausibly contribute to reduced age-related thermogenic adipose tissue activity. Conversely, IL-6 circulating levels increase with aging (Maggio et al., 2006; Kwon et al., 2020), although the contribution of skeletal muscle for age-related changes is not established.

### 4.5 Immune cells and inflammation

Aging is associated with a systemic pro-inflammatory state, so-called ‘inflammaging’, which poses a high risk of metabolic disease, disability, frailty, and premature death (Ferrucci and Fabbri, 2018). Inflammation and adipose tissue dysfunction are closely linked in a vicious cycle and negatively affect the risk of metabolic diseases (Kawai et al., 2021). Both processes are observed during aging, and the effect of many immune cells and products involved in adipose tissue inflammation with aging has been characterized (Ou et al., 2022). Emerging evidence indicates that this relation also affects thermogenic adipocytes and can play a role in diminished age-related nonshivering thermogenesis.

Pro-inflammatory cytokines were shown to induce brown adipocyte apoptosis directly (Nisoli et al., 2000), impair brown adipocyte activity and beige adipogenesis (Bae et al., 2014; Villarroya F. et al., 2018), and suppress cold-induced thermogenesis (Goto et al., 2016). The exact mechanisms underlying these effects are not fully understood but may involve direct actions to downregulate the expression of thermogenesis-related proteins (Rebiger et al., 2016) or be mediated by suppression of β-adrenergic signaling in adipose tissue (Goto et al., 2016).

Findings from a study involving a murine brown preadipocyte cell line indicated that mature brown adipocytes exhibit increased expression of enzymes related to antioxidative defense when compared with preadipocytes. However, exposure to inflammatory cytokines such as tumor necrosis factor-alpha or interleukin 1β decreased antioxidative enzyme expression, which was accompanied by increased reactive oxygen species production and down-regulation of thermogenesis-related proteins, such as UCP1 and β-Klotho (Rebiger et al., 2016).

It was reported that aging upregulated genes related to catecholamine catabolism in mouse visceral adipose macrophages in an NLRP3 inflammasome-dependent fashion, leading to decreased lipolysis (Camell et al., 2017). Although the study did not assess thermogenesis, it is possible that increased catecholamine degradation with aging also occurs in adipose tissue depots in which thermogenic adipocytes are found and could, hence, affect their function. There is also emerging evidence for the role of other adipose-resident immune cells in age-related reduction in thermogenesis. Type 2 innate lymphoid cells within adipose tissue were shown to decrease and display functional abnormalities in aging that were directly related to impairment of the response to cold (Goldberg et al., 2021).

A recent study investigated the transcriptomic signature of 17 organs and plasma proteomics across mouse lifespan and found widespread activation of immune cells with age, first detectable in white adipose tissue depots at middle age (Schaum et al., 2020). Such findings further support that adipose tissue-resident immune cells may account for reduced recruitment and activity of beige adipocytes with aging, given that this cell type arises in white adipose depots.

## 5 Implications of thermogenic adipocyte loss with aging

Overall changes in adipose tissue mass, distribution, and function with aging have a significant impact on other tissues, given the widespread crosstalk between adipose tissue and other tissues through several pathways, including those involving metabolic fuels, endocrine factors affecting insulin sensitivity, the inflammatory response, and circulating miRNAs (Ou et al., 2022).

In particular, the implications of age-related changes in thermogenic adipocytes are not fully understood. It is well-acknowledged that brown and beige adipocytes’ diminished mass and activity impairs the response to cold and increases the risk of cold-induced hypothermia in the elderly. Moreover, decreased energy expenditure may contribute to reduced energy consumption in the elderly (Ou et al., 2022).

There is increasing evidence that loss of thermogenic adipocyte activity with aging affects metabolic health. Brown and beige adipocytes act as a metabolic sink for glucose and lipids, so the age-related impairment in their function may contribute to an insulin-resistant state and metabolic disease risk (Chondronikola et al., 2014; Cohen and Kajimura, 2021). It is also possible that the secretion of brown and beige adipocyte-derived factors diminishes with age and may, in turn, negatively affect metabolic homeostasis. This remains to be explored and appears not to be broad since the secretion of some factors from brown adipose tissue, such as the exercise-stimulated lipokine 12,13-dihydroxy-9Z-octadecenoic acid, do not change with age (Stanford et al., 2018).

## 6 Targeting thermogenic adipocytes to improve health span

Many strategies to improve health span or even extend lifespan have targeted the adipose tissue, with promising results (Ou et al., 2022). Most of them have focused on white adipose tissue, in which beige adipocytes arise, but some recent evidence suggests that thermogenic adipocytes may also be addressed to improve age-related diseases, such as insulin resistance, type 2 diabetes, nonalcoholic fatty liver disease, and cardiovascular diseases, and even increase longevity (Zoico et al., 2019).

The advances in understanding the mechanisms underlying thermogenic adipose tissue aging have provided promising targets and strategies to improve health span. For example, alpha-lipoic acid supplementation restored mitochondrial lipoylation in adipocytes of aged mice, enhancing the ability of brown adipose tissue to uptake glucose and execute thermogenesis, implying that the age-associated decline in brown adipose tissue function is potentially reversible (Tajima et al., 2019). It is currently unknown whether this approach would also impact beige adipocytes, and this is important to be defined given that the latter are the predominant thermogenic adipocyte in adult humans.

Another essential question to be addressed is the effectiveness of such strategies during adulthood. Overall, the decline in the ability to recruit beige adipocytes is apparent by the fourth decade of life in humans (Yoneshiro et al., 2011) and is also observed in rodents (Rogers et al., 2012). Such findings may be accounted for, at least in part, by the diminished beige APC pool size and its impaired differentiation ability (Berry et al., 2017). Therefore, it would be of uppermost importance to establish the mechanisms underlying the involution of brown adipose tissue and reduced beige and brown APC pool to unravel novel pathways that could be targeted to preserve or restore it. Such pathways most likely involve genetic and environmental factors, so this could be accomplished by examining individuals with unexpectedly high occurrences of thermogenic adipose tissue at a higher age (Vosselman et al., 2014).

Some pathways determining APC pool have also been identified, such as the one involving *FSTL1* repression (Huang et al., 2022). Interestingly, an open-label phase 1 pilot study assessing the short-term effects of the senolytics dasatinib and quercetin in subjects over 60 years with chronic kidney disease older showed that they reduced adipose tissue senescent cell burden and senescence-associated secretory phenotype in adipose tissue (Hickson et al., 2019). Whether this would translate into increased ability to recruit thermogenic adipocytes or improved health span or longevity is unknown, but the findings point to the potential to diminish the senescence phenotype associated with reduced APC pool size.

Various dietary interventions comprising nutrient or overall energy restriction, such as caloric restriction (CR), intermittent fasting, and dietary restriction, have favorable effects on health and increase longevity (Kennedy et al., 2014). Caloric restriction (CR) is a long-known strategy to extend life span and improve health in multiple organisms (Madeo et al., 2019), with profound effects on adipose tissue distribution and function (Das et al., 2004). This suggests that adipose tissue changes may be an important mediator of the benefits of caloric restriction and is further supported by partial recapitulation of CR effects by surgical removal of visceral adipose tissue (Muzumdar et al., 2008). There is evidence from preclinical studies that age-related dysfunction of thermogenic adipocytes is improved by CR (Valle et al., 2008a; Rogers et al., 2012), and that thermogenic adipose tissue is directly linked to the metabolic benefits of intermittent fasting (Li et al., 2017) and protein restriction (Dommerholt et al., 2021). However, whether thermogenic adipocytes are implicated in the life extending effect of CR is still to be determined.

Genetic mouse models have provided invaluable insights not only for understanding thermogenic adipocyte recruitment and function but also for identifying potential targets to improve thermogenic adipocyte function to increase longevity. A classical model of mammalian longevity and increased health span is the growth hormone (GH) receptor knockout mouse (Coschigano et al., 2000), which exhibits an enlarged interscapular brown adipose tissue depot and increased expression of UCP1 and FGF21 (Li et al., 2003). Conversely, transgenic mice overexpressing GH are short-lived and present lower UCP1 expression (Bartke, 2003). These findings point to the inhibitory action of GH signaling on thermogenic adipocytes and suggest that they may contribute to extended lifespan in the GH receptor knockout model of longevity.

Transgenic mice overexpressing phosphatase and tensin homolog deleted on chromoson 10 (PTEN) exhibited increased longevity, which was accompanied by decreased adiposity, improved glucose tolerance, increased energy expenditure, and hyperactive brown adipose tissue activity (Ortega-Molina et al., 2012). Similarly, the regulator of G protein signaling 14 (RGS14) knockout mice showed extended lifespan, metabolic health, and improved response to cold. Surgical removal of brown adipose tissue reversed the latter phenotype, indicating the direct involvement of thermogenic adipose tissue (Vatner et al., 2018). Pathways such as those involving GH, PTEN, and RGS14 signaling could be addressed by pharmacological tools or maybe gene therapy to improve health span or even longevity.

## 7 Conclusion

The interest in understanding thermogenic brown and beige adipocyte biology was fueled in recent years due to their identification in adult humans and delineation of their role in whole-body energy homeostasis. Data from rodent models and human studies have pointed to age-related changes in thermogenic adipocytes and indicated their role in disease development. Increasing understanding of the mechanisms underlying thermogenic adipocyte dysfunction in aging has identified some pathways targeted to increase health span and longevity in rodent models, and many more may be discovered soon. Further studies should address how these findings can be translated into human physiology.
